# Advocacy Science: Explaining the Term with Case Studies from Biotechnology

**DOI:** 10.1007/s11948-017-9916-0

**Published:** 2017-06-08

**Authors:** Ksenia Gerasimova

**Affiliations:** 1 Centre of Development Studies, University of Cambridge, Alison Richard Building, 7 West Road, Cambridge, UK; 2 Higher School of Economics, Moscow, Russia

**Keywords:** Biotechnology, GM crops, NGOs

## Abstract

The paper discusses the use of term ‘advocacy science’ which is communication of science which goes beyond simple reporting of scientific findings, using the case study of biotechnology. It argues that advocacy science should be used to distinguish the engagement of modern civil society organizations to interpret scientific knowledge for their lobbying. It illustrates how this new communicative process has changed political discourse in science and general perception of the role of science in contemporary society.

## Introduction

With the discovery of DNA and ability to separate and transfer genes from one organism to another, scientists have created a new way of solving many tasks that were unimaginable in the past. With this new tool, however, scientists have also found new challenges that could hardly be conceived in the earlier days such as the need to communicate and advocate their research to the public and to policy-makers, secure funding for expensive experiments, and address the ethical concerns resulting from their work. The advent of biotechnology is rather an extreme example of how science has had to confront all these issues, but the intense debate and political lobbying that relates to the science make it a good case for discussing how the place and role of science in society and its political discourse have recently changed.

This paper picks up on one feature that can be found in these processes, advocacy science. Advocacy and objectivity of science is a popular topic, but the term ‘advocacy science’ itself is relatively little discussed, although it does appear in the scientific debates. In 2011 the National Science Foundation even held a two day workshop on advocacy science’s important implications for the relationship between science and society as well as for public policy decisions. The American Association for the Advancement of Science (AAAS) understood advocacy in science as ‘scientists ‘doing’ advocacy’ and ‘increasingly being encouraged by people inside and outside science to become engaged with the public policy process’ (Runkle 2012, p. 1). The existing literature touches upon advocacy science, for example Grundmann (2011) uses the term in his discussion of bias in climate change politics. Steve Pierson (2012) from the American Statistical Association argues that there are two categories of science advocacy – policy for science and science for policy which are used to inform policymakers and advocate certain decisions. The latter opens a place for political alternatives offered by groups that were previously little involved in policy-making processes, such as social movements (Harries-Jones 1991). However, it might also create political opportunism to cover up ‘poor science’, on one hand, and, on the other, to promote political decisions which would otherwise be supported by only marginal, off mainstream groups. While many natural scientists, as will be shown from the examples below, continue to believe that science is purely about producing knowledge, advocacy science goes beyond that: it understands the growing importance of interpreting knowledge and of making political lobbying out of created knowledge in modern politics. Members of the AAAS workshop reported that scientists become engaged in advocacy, and have recommended putting ethical standards in place by implementing “guidelines for advocacy” (Runkle 2012). They have recognized that advocacy science is about the communication of scientific results by scientists and goes beyond reporting and explaining to advocating’ (ibid.). Professor Nielsen (2001), himself a natural scientist with a public role, has distinguished the professional activities from the personal values and interests of scientists and recommended to avoid mixing science and advocacy. This becomes an issue particularly ‘when there is both uncertainty about facts and disagreements about values’ (Sarewitz 2012).

Recently, however, it is difficult to avoid mixing science and advocacy, since advocacy, as pointed out by the AAAS, is a part of a global process: it is ‘a condition not of behaviour of individual scientists, but of the political and institutional context for science’ and scientists have to adapt to changes in the context in which they operate.

This paper generally agrees with the conceptualization of advocacy in science offered by the AAAS, and suggests discussing the term ‘advocacy science’ as a recent socio-political phenomenon which can be illustrated by the case study of biotechnology, where ‘uncertainty about facts and disagreements about values’ are both present.

Another recent characteristic of the process is the involvement of civil society and its groups and movements in advocacy science. Nongovernmental organizations (NGOs) are active in the climate change debate and its policy-making (Grundmann 2011) and in biotech. It is widely recognised that NGOs, particularly in Europe, are responsible for blocking the commercialization of genetically modified (GM) crops. Here it is easy to equate advocacy science with NGOs engagement in science to lobby against mainstream science resulting in accusations stigmatizing the anti-GMO movement. Perhaps it might be more helpful to discuss how the mixing of professional skills and personal values occur in contested scientific topics such as GM plants and what this means for modern scientists and for civil society’s discourse in science as a result.

An obvious method to analyse political discourse would be critical discourse analysis which is ‘a type of discourse analytical research that primarily studies the way social power, abuse, dominance and inequality are enacted, reproduced, and resisted by text and talk in the social and political context’ (van Dijk 2001, p. 352). In the case of political discourse, it is critical political discourse analysis (CPDA) that addresses the discursive conditions and social and political consequences resulting from the power distribution within the political discourse studied (van Dijk 1995). ‘Political’ refers to the field of politics, which includes political actors, the recipients of political communicative events, including the general public, and political structures and institutions (van Dijk 1995, pp. 12–13).

According to Fairclough (1995), who, among others, mastered and promoted the use of critical discourse analysis (CDA) in political studies, there are three levels of CDA: textual analysis, interpretation (processing analysis) and explanation (social analysis), which appears as inductive reasoning. This paper, however, implements elements of CPDA, but with a deductive logic and with a reference to the institutional theory. First, it briefly describes the institutional context in which modern science, including biotechnology exists. Then it identifies two general discourses in the political discussion of biotechnology (corporate science and citizen science). And finally, it analyses two cases of biologists, A. Pusztai and G.E. Seralini, which are argued to represent cases of ‘advocacy science’ in biotechnology leading to significant political implications in the banning of GM crops.

## Science and Changes in the Modern Institutional Context

There can be other different approaches to what science is, yet it is possible to use them all complimentarily and accept that science is about generating theoretical knowledge and its practical use (Derry 1999). The functions of science in society include creation of knowledge, communication of its value and promotion of further application. From a pragmatic perspective, science challenges traditional beliefs and invents new technologies which can be used for benefit or harm. Consequently, the introduction of these technologies brings changes in social organization and new values. This is how science produces an impact on society, as explained by Russell (1952).

For many centuries early science was elitist and relied on support from the rich and those in power. Since the eighteenth century, with cessation of persecution of science and more acceptance of freedom of thought, science started to develop its modern, more pragmatic or what Russell has called ‘mechanistic outlook’ (ibid., 15). In the accord with the Russell’s argument about the link of science to industry and war, in the twentieth century, in the period between the two world wars and after, science has come to the close attention of policy-makers who did not want ‘the progress of science no longer to be left to chance’ and recognised the need of scientific work for the solution of practical problems (Salant 1920).

The result of this additional attention, on the one hand, meant secularization of science, and, on the other, politicization of science. Although science has always been political, external influences on science have existed even before the Western scientific revolution (Weinberg 2016, p. 253). What has changed was a new conception of science as ‘impersonal, without room for supernatural interventions or (outside the behavioural science) for human values’ and ‘with no hope for certainty’ (ibid. p. 254). But science is still political. For example, scientists may, only at the beginning of their career particularly, naively believe that genetics, such as ‘population genetics, studying fruit flies and so on, [have] had absolutely no consequences for human socio-political issues’ (Lewontin 2008, p. 3). Obviously, they soon come to the conclusion from their own experience that science, and particularly, genetics is political. Too much involvement of the state, particularly an authoritarian one, can lead to ideological and methodological distortions in science. The Nazi eugenics and Lysenkoism are such examples.

Knowledge, in relation to technology has become such a powerful tool to win supremacy in the international realm that ethical issues have become overshadowed. An extreme example of that is the case of the Japanese microbiologists who gave their notebooks with the results from their experiments testing potential bacterial weapon on prisoners to the Americans in return for their amnesty after the Second World War (Muller Hill 1998).

Development of more open systems of knowledge has created complexity and raised the issues of uncertainty and unpredictability. After 1945 predictability and control became ‘hallmarks of an accomplished modernization arrogantly characterised by assertions of universalism, openness, rationality and efficiency’ (Nowotny et al. 2002, p. 6). Science and technology enjoyed good reputation and funding was abundant. But this did not last long. Consequently, the controlling imperatives of the welfare state were shaken by such events as the oil crises of the 1970s and the collapse of the Soviet Union. As ‘the (assumed) regularity of society and the predictability of progressive science’ were lost, a new confrontational discourse came to the fore (ibid). The epoch of the ‘risk society’ had arrived before Beck (1992) coined the term, as it became clear that one mistake can bring costly results, as it happened at Chernobyl. Scientists themselves, such as Andrei Saharov, could not help avoiding ethical considerations over the possible misuse of their discoveries. Under such high stakes, science had to become more reflexive and public policy employed a precautionary approach in an attempt to manage uncertainty and possible risks of scientific research and its outcomes. In many cases, such as in the PCB (polychlorinated biphenyls), DES (synthetic oestrogen diethylstilboestrol) and BSE (bovine spongiform encephalopathy) issues, risk management by public authorities was not adequate and distorted communication of scientific knowledge (Harremoes et al. 2002).

In parallel, other institutional actors, such as business and civil society (the third sector) started to act as alternative sources of commissioning and managing scientific research. Limited public funding is one explanation of why much of biotechnology research is outsourced to business (Kingsbury 2009) which, in return demands an industrial application of the funded research and guarantee of a financial return. Thus, if in the twentieth century science was mainly pressurized by the state to solve political challenges, in the twenty-first century it is pressurized to provide market-based solutions by business.

Another important institutional trend, in parallel to the loss of the supreme role of the state,^1^ as argued by transnational theory, is the expansion of civil society. The processes (reduction of censorship, control by the state and ties with the church) that developed modern science, also contributed to the development of the civil society in the Western world in the eighteenth century (Weber 1962, p. 3). Civil society or third sector started to blossom from the second half of the twentieth century: from around 500 organizations in the 1960s mainly in Western Europe and the USA (Feld 1972, p. 182) to millions of organizations worldwide today. Skjelsbaek (1971) has found a correlation between the level of economic development, technological progress of the society and the concentration of NGOs in the society. Finally, the nature of the third sector organizations, NGOs has been transformed. From self-help informal groups they have grown into professional organizations with paid staff and have now obtained a new role of vox populi, often without formal democratic recognition and claiming to represent the rest of community, yet refusal to collaborate or disagreement with them will be viewed as an attack on democracy (Narochnizkaia 2008). It can be argued that NGOs, although frequently now being multi-national organizations with major political power and influence and large budgets, are unelected, unrepresentative and often unaccountable to the general public, as well as being unregulated except as charities or companies when they are registered.

New actors become actively involved in the discussion on the role of science and its policy-making. As a result, the perception of science as linear from scientific discovery to useful application has been changed to ‘outcomes of social networks that incorporate a wide range of social actors’ (Bora and Hausendorf 2010, p. 2).

It is possible to suppose that the more complex institutional structure provides more opportunitiess for new institutional actors to become involved. For example, the European institutions, a modern post Second World War complex institutional arrangement, faces cross-sectorial pressure in the area of GM agriculture. Despite the attempts to overcome the fragmented departmental nature of governmental policy making, the EU institutions have become advocacy arenas for gaining control over the regulatory processes of the new technology (Lewidow 2010).

According to Russell (1952), science changes society. But perhaps society also affects science. It is possible to argue that the process goes both ways. The case of genetics and its social ideology of eugenics is a good example. Although initially inspired by the humanistic cause to decrease child mortality (Bashford 2015), eugenics have gained a bad reputation after its misuse by the Nazi regime which led to inhuman cruelties. After the Second World War, German society developed a traumatized collective memory which led to the suspicion of other applications of genetics, including transgenic plants (Muller Hill 1998; van der Heijden 2010). In German-speaking states, GM crops are banned. Public suspicion of genetic engineering has directly affected the advances of biotechnology; it has questioned and added a more general scepticism about the goals and the social impact of science and technology as a whole (Davies 1991, p. 8, cited in Turney 1998).

Thus, one can argue that institutional transformations in general and those closely associated with science have changed the general discourse of science in society, its episteme, at least so it appears in the case of biotechnology. And this takes place through the communicative interaction of different social actors.

According to Habermas, communicative action in society is such an action in which social actors seek common understanding through a rational argument in a practical political discourse, in which the validity of norms could be accepted by everyone. Practical reason provides justification for the universalistic and egalitarian concept of morality, law and science (Habermas 1984). These days, however, the universal approach is being contested by the postmodernist conceptation of political discourse as a contest of local narratives (Fairfield 1994).

An illustration of the collision of these two views can be found in the discussion between natural and social scientists. Professor Muller-Hill, the German chemist and geneticist who specialised in protein-DNA interaction and gene control, calls himself ‘an active scientist’, since he started to discuss the application of science in society, reminding the Germans about their painful past with genetics. He complained about the socio-anthropological research by Latour, Woolgar and Knorr-Cetina who defined science as a social construct, which meant that ‘they say science evolves under the pressure of various social groups either in this or in that direction’ and it can be found in all branches of science’ (Muller Hill 1998, pp. 40–41).

Muller-Hill disapproved of their socio-anthropological research and arrived at the conclusion:

They watched the men and women in the white coats and noted their strange habits. They were uninterested in the theories the scientists studied. They did not try to understand the logic of experiments the scientists performed. They simply watched their bizarre behaviour. They noted that often the chief, mostly a male, determined the direction of the research. He got the money. They thought they had observed that the chiefs, as the highest shamans, determine the actual structure of the scientific theories and the outcome of the experiments (ibid. 41–42).

For him, by calling science a social construct, social scientists denied that science delivered the truth, since ‘science without truth is no science’ as it is based on experiments, and discoveries. Under the definition of social construct, results of scientific studies can be arbitrary and thus intentional misinterpretation of experiment would not be considered as fraud (ibid. pp. 42–51).

And yet, even within one natural science discipline, biology, there are two confronting discourses – molecular reductionism and ecological holism (Looijen 2000). This debate is not about methodologies and different schools of thought, but is a fundamental epistemological one. For example, one of the determinators of structure of the DNA molecule, Francis Crick, radically argued that an organism was only a collection of atoms and molecules (Crick 1966). Such an approach means that scientists can also assemble any life-form dependent on such molecules in a laboratory. On the contrary, proponents of ecological holism or nature fundamentalism - such as Dr Florianne Koechlin see an organism as more than a sum of molecules and argue for the supremacy of context in its development, and with a variety of functions. The definition of genes is seen as elusive as they are part of a dynamic network (Koechlin 2005). Some reductionists have even changed their opinion, as Arthur Peacocke:

I concluded that in many important instances the concepts and theories that constitute the content of the sciences focusing on the more complex levels are often (not always) logically not reducible to those operative in the sciences that focus on their components. Sometimes a variety of independent derivation, identification or measurement procedures directed at a particular complex level fund an invariance in the concepts and referential terms of the theories needed to account for the phenomenon associated with them (Peacocke 1996, 197).

Interestingly, what may sound like an ideological and epistemological debate has led to practical implementations: inclination of funding support towards projects in molecular biology and reductionism (ibid. p. 196).

The enhanced variety of social actors involved in the discussion of science in society provides a diversity of opinions, but it does not seem to relate them to each other. In many cases it appears as if there is a postmodernist view of science, when interpretation of knowledge becomes more important than just the creation of knowledge.

## Different Kinds of Science

The previous discussion of the modern institutional context has identified the increased engagement of two institutional actors, business and civil society, in the social management of science with the decreasing role of the state in the background. These two actors have produced their own science discourses which are worthy of further study.

The business sector has been brought into scientific research due to the capital-intense nature of modern science, although business has always been interested in science in order to use scientific results for commercial purposes. That, in its turn, has resulted in changes in the nature of science: academic science started to be replaced by industrial science (in the 1960s) and post-industrial science oriented to problem solving in local contexts (Ziman 1996). The difference between industrial and post-industrial sciences is in substitution of ‘market competition’ by ‘command management’ by a small group of multinational companies (ibid. p. 76). Both led to the establishment of post-academic science which adopted postmodern philosophy, as feared by some scientists such as Muller-Hill as shown above. The spread of transnational corporate science, which is a ‘business-science hybrid’ has also raised issues of public interest and regulatory regimes for intellectual ownership of research property (Glover 2002). The corporate R&D laboratories conducted industrial research and produced new knowledge, so it is far too simplistic to see them ‘as university research laboratories in exile as instruments of big business that manipulate once-pure scientists for corporate ends, or as second-rate research institutions’ (Hounshell 1988). Corporate management of research providing the basis for continuity of developing and maintaining laboratories, was able to balance short term needs with long term strategies in research which then led to decentralised research (ibid.).

Some products developed by corporate scientists served well. Even DDT (dichlorodiphenyltrichloroethane), the product of Geigy, was developed to fight against insects and was useful in the World War II to address sanitary and hygiene limitations and proved to be an effective insecticide in agriculture until it was found toxic to the environment (Mellanby 1992). The DDT case shows that even proven results might be contested in the future. The Haber–Bosch process of synthesizing ammonia developed at Badische Anilin und soda Fabrik (BASF) allowed to fix nitrogen in soil and grow more crops. Smil argues that ‘without this, almost two-fifths of the world’s population would not be here’ (Smil 1999).

Corporations also provide funding to universities, and this gives ground for activists to accuse academic scientists of biased research. Natural scientists may find discomfort in this new expectation of not only making discoveries but by providing socially acceptable interpretations of these discoveries, lobbying for their further use and being aware of their public image. However, if scientists are not ready to operate in this new mode, they can be threatened by losing the ability to conduct their research altogether. For example, Sir David Baulcombe, Head of the Department of Plant Sciences at the University of Cambridge, had to speak in front of farmers in the county of Norfolk in the UK. While he was prepared to explain the scientific aspects of his research, he was confronted by the angry audience emotionally stimulated by an anti-GM activist (Baulcombe 2014). It was a traumatic experience, but does that mean that academic scientists should avoid corporate funding? It is hardly possible. While universities continue to receive corporate funding and there are issues of corporate ownership rights over technologies (for as long as patents last), academic researchers are also involved in open technologies. For example, in the OpenPlant project, while there is still a need to protect the intellectual property of applications with potential commercial applications, its creators allow ‘a family of generic lower-level tools that are largely free of IP constraints’ to be freely shared to promote innovation in plant synthetic biology (OpenPlant 2016).

Simultaneously, civil society has also become more involved in doing and discussing science. For example, a multinational NGO, the World Wildlife Fund (WWF), has run its own research programs aimed at the conservation of species threatened with extinction since 1962 (WWF 2015). They often commission research by individual scientists and academic institutions (Schwarz 2010). In the WWF specific scientific activities are accompanied with political lobbying and education of the general public, and the organization enjoys its own pool of financial support based on mixed sources of funding. Increased interactive science communication with the public has been documented in another new concept – citizen science (CS) (Irwin 1995). In certain cases some academic research projects benefited from including extra human and financial resources provided by concerned citizens. For example, monitoring of changes in migration of monarch butterflies in the USA was framed into a network of projects run by volunteers with financial support from different foundations (Howard and Davis 2015). While for some authors (Cohn 2008) it seems natural to benefit from inclusion of volunteers into science research, others are more critical about its implications for academic science. For example, Riesch and Potter (2014) argue that ‘overly grand aspirations for CS’ can be misleading. Individual projects can illustrate benefits of spreading knowledge, but on a systematic level there will be a number of concerns of an ethical character, such as research authorship and even fewer paid opportunities for professional scientists.

The promotion of citizen science brought a closer interaction of professional scientists with lay, non-academically qualified members of society to discuss science and make decisions over its applications. This has been reflected in the language and style in which these discussions are being held. As it has already been shown in the example of Professor Baulcombe, in the debates between scientists and lay people about biotechnology and its GM products rationality sometimes started to be opposed to emotionality. Here is another example:

I went to a panel at the nearest high school with a green member of the state parliament. There were 500 people in attendance and it was packed. I was winning the argument, and suddenly (his opponent started to scream and cry. So I said to her, “don’t you think we should stop being so emotional and be more objective/factual about this?” At that point a 50 year old lady in the audience stood up and said, “Mr X., are you only a brain or do you actually have a heart in this issue, too?’ That’s when it became very clear to me that…the problem for the big corporations is that they are already anonymous and faceless, perfect target for activists, you can’t win with the rational stuff, have to show a human face (Rao 2009, p. 160).

Some members of the biotechnology community have recognized the current winning by the anti-GM crops lobby in the debate (Schurman and Munro 2010). Thus, one may argue that lay people seem to win the debate, at least in the case of the GM crops, by better knowledge of public relations and of playing on instincts and emotions, particularly fear, in the public. The apt choice of rhetoric appropriate for a specific local context has been crucial.

Framing the GMO debates in the Frankenstein rhetoric proved to be a decision which worked for the anti-GM crops lobby. Today the term ‘Frankenstein science’, named from a famous short story by Mary Shelley, refers to crazy, ill-fated scientific experiments leading to destruction, inaccurate science or pseudoscience.

The linking of GMOs with Frankenstein’s creature was really just a matter of time. In 1977 Arthur Lubow in the New Times newspaper article raised concerns about ‘modern Dr Frankensteins have found a way to create brand-new forms of life’ (Lubow 1977). Once the debate on GMOs focused on food, being set in the context of the bovine spongiform encephalopathy (BSE), commonly known as mad cow disease crisis in the 1990s, a new term Frankenfood appeared. The term was coined by Paul Lewis, Professor of English at Boston College in his letter to the New York Times in 1992 (Casetta and Tambolo 2013).

The debate was then joined by Jeremy Rifkin, a founder of an NGO Foundation on Economic Trends (FOET) and an American activist, economist by training, who also consulted the European leaders at the highest level (FoET 2013a). He led one of the first anti-GM crops campaigns called the Pure Food Campaign (PFC). This campaign opposed the commercial arrival of GM food, the GM tomato Favr Savr by Calgene, the first ever GM food product to reach consumers. Rifkin’s initiative brought together ‘a coalition of organic farmers and restaurateurs, consumer and environmental groups, and animal welfare organizations opposed to the use of genetic engineering in food’ (FOET 2013b). The campaigners distributed flyers featuring a dinosaur pushing a grocery basket labelled “Bio-tech Frankenfoods.” Their message was that “corporate science” could alter and create life forms with “enormous and frightening” possibilities (Hamilton 1993).

In his books Rifkin recognized the strong power and ‘the “terrible nature” of ‘new science’ represented by biotechnology to bring enormous changes to society and questioned such consequences including such ‘monstrous and unwarranted intrusion’ in human lives (Rifkin 1999, p. XII). He spoke about potential risks for human health and the environment from the new technology and elaborated the anti-corporate argument, especially its ownership aspect, by portraying business as gene-hunters trying to monopolise indigenous plant knowledge and being confronted by ‘good guys’ from NGOs. But he also touched upon the scientists whose faculties received corporate funding (ibid).

The Frankenstein rhetoric served well to present the role of science in developing new technologies. The key concepts were the ambivalence of modern technology and its uncertainty (Turney 1998). Some have argued that with such a metaphor the comparison of biotechnology with the Frankenstein story has done harm to the public image of technology as it was not ‘helpful in understanding and lead to further astray’ (Casetta and Tambolo 2013). The fate of the genetically engineered tomato in both sides of the Atlantic Ocean was similar, as it was withdrawn from production and availability by the activities of consumers.

According to Belinda Martineau, one of the Calgene biologists, during the hearing over Favr Savr there was ‘not much meaningful listening’ and almost no ‘meaningful exchange’ between the two sides (Martineau 2001). As Martineau reflected upon the beginning of the debates, she recognized the poor ‘execution (or lack thereof) of bringing that information gap’ to the opponents and general public by the pro-biotech camp’. In their explanations they used oversimplification, conveyed general and not so convincing ideas such as ‘no evidence of any of these products is unsafe’ and did not refer to scientific publications (ibid.). No wonder, they could not win public opinion.

The other successful tactic of the anti-GM crops movement is mimicry of scientific activities. This includes involvement of people with a scientific background and production of reports that have the features of scientific reports, yet they are tailored for non-specialist readers, written by activists and deliver a specific political message.

An example of such reports are publications produced by the Open Earth Source, a British anti-GM crops NGO (Antoniou et al. 2012, 2014) by Dr Michael Antoniou, a biologist employed by professional science institutions. While Dr John Fagan holds a PhD in biochemistry and molecular biology from Cornell University, USA. In 1994 he took a stand against genetic engineering, renounced his research grants and decided to dedicate his life to anti-GM crops activism (Fagan 2007). Alongside them is a social scientist Claire Robinson, with a Master degree, in which field is specified neither in the reports nor at the website.^2^ This led some inquisitive readers to question their credentials (Griekspoor 2014).

At the same time the pro-GM crop side also involves people who are not all natural scientists by training. Mark Lynas, a former Greenpeace volunteer, who turned into a supporter of GM crops. He ‘had not read a single scientific paper on the subject’ until his public coming out for GM crops (Forbes 2011). Since then he has published a number of books, including ‘The God Species’ which has received positive reviews particularly for his factual knowledge and emotional presentation, and has consulted governments on the matter he wrote about (ibid.). He is not seen as a scientist by scientists or by activists.

The boundaries between science and lay people have been blurred. In the papers written by activists the academic format which one may call heavy reading, is replaced with a style suitable for mass readership. The message communicated though is definite and clear and requires certain political actions from its readers (i.e. to ignore and protest against GM food, or protest against pro-GM lobby).

Activists’ science policy activities also mimic public democratic actions, such as public consultations. This raises questions of representation and public interests. An example of an alternative participatory exercise is the People’s Report on GM crops, based on separate juries that met in Hertfordshire and Tyneside in the summer of 2003 and deliberated on the issue of GM crops. They were organised by a team from the Policy, Ethics and Life Sciences Research Institute (PEALS), University of Newcastle, which had been inspired by the UK government GM debate was held in 2002. The report was presented as an alternative for the official debate and ‘condemns’ ‘the way in which the elected Government has merely paid ‘lip service’ to public debate on such a major issue as GM’ (PEALS 2003, p. 2). The jury had funders, such as the Consumers Association, Greenpeace, the Co-operative Group and Unilever. Given the long standing position of Greenpeace to promotion of organic agriculture and opposition to GM crops, it is not surprising that the conclusions of the report list ‘a critique of current conventional agricultural practices based on high inputs of fertilisers and pesticides’; ‘a proposal for support systems for agricultural techniques that do not rely on artificial chemicals, such as organic farming; ‘a call for incentives to encourage retailers to act in the interests of smaller and organic UK farmers, rather than to import food from abroad’; and ‘call for bodies that regulate new agricultural and food technologies to be made fully accountable to citizens, together with specific proposals for reform’ (ibid).

This leads us towards a general discussion of what these civil society groups are. Much has been said about the bottom-up approach and civil society activism, such as by self-help groups (Edwards and Fowler 2002). However it is also true that there are organizations with a supposedly non-profit nature and contrasted to the business sector that are run by professionals making money out of conducting certain lobbying activities. They might claim to act in the interest of humanity, but what is the common good and how it should be lobbied for is decided by a small group of people, not even by a large pool of the organization’s volunteers. Greenpeace is an example of this situation (Zelko 2013).

As shown above, in the postmodern context science has been fragmented into different sciences: academic science, corporate science, citizen science and activist science. As it becomes difficult in many cases to provide definitive answers, particularly to the long term consequences resulting from the application of research, which demands time, space and resources for the further studies, they are used against each other to try to win over the opposition in what are actually political campaigns.

## Case Studies of Advocacy Science

### Case Study 1: The Puzstai Case

Dr Arpad Puzstai, a Hungarian biologist, spent most of his academic career working for the Rowett Research Institute in Aberdeen and his main area of expertise was plant lectins, toxic substances naturally produced by plants to repel insects (Pusztai 1991).

In August 1998 he appeared on a Granada Television program called ‘World in Action’. In that public appearance he felt it necessary to raise and share his concerns over GM foods, in regards to the study conducted at the Rowett Institute aiming to transfer a snowdrop plant gene to potato. From his much later interview in The Guardian newspaper, it appears that he was not prepared to deal with the complexity of mass media and possible consequences of making a strong claim about GM plants: ‘I am an academic scientist. I’ve never been exposed to this…I’m really not a very media person’ he said (Randerson 2008). The director of the Rowett Institute did not foresee the coming storm either, and he even called on his subordinate colleague to congratulate him ‘on the modest way in which he had presented the evidence on the programme’. The original study to which Pusztai referred in his interview was a comparison of the effects on rats from eating GM potatoes with lectin transferred from the snowdrop and non-GM potatoes (ibid.). In the interview he referred to the limitations of testing procedures and stated “I find it’s very unfair to use our fellow citizens as guinea pigs’ (GM-Free 1999, p. 4).

Suddenly, the media and public authorities took up that study. The stories in the media added to confusion by referring to potatoes modified with a lectin transferred from jack-bean that is poisonous to mammals. Interestingly, it is not possible to trace the original source of this to the media (Randerson 2008).

Pusztai’s boss, Professor Philip James, who worked with the British government during the BSE crisis and produced a blueprint document outlining recommendations on how to regulate food safety issues, had to intervene and to speak up for the reputation of the institute and address the storm in media. The Institute issued a press release and its head who was Professor James criticized Pusztai: ‘My change in attitude was dramatic because I discovered that Pusztai…had never conducted the studies which he had claimed’ (ibid.). The Institute published an audit report. In response, Pusztai strongly denied this accusation, but nonetheless he lost his job at the Institute.

The immediate discussion of the Puzstai study was then followed by what the opponents of GM crops called the ‘campaign of disinformation’: ‘Faced with this onslaught on its policies, Tony Blair’s government sprang to the defence of its biotech associates with a stepped-up campaign of disinformation’ (GM Free 1999, p. 5). The anti-corporate rhetoric accusing the Rowett Institute of receiving £140,000 grant from Monsanto was also noted (ibid. p. 4). In the scientific circle the affair produced a ‘titanic battle of experts’ (Fedoroff 2011). First, Puzstai sent the audit report and his TV interview script to a number of scientists, who in their turn issued a memorandum in his support aiming to ‘remove the stigma of alleged fraud’ and to restore their colleague’s scientific reputation (Lee and Tyler 1999). Some, such as Professor Pierzynowski stressed that he did not find the audit report objective and that the whole incident was ‘per se, dangerous, not only for Dr. Pusztai, but generally for free and objective science’ (Fedoroff 2011).

In October 1999 The Lancet published an article by Pusztai and Ewen which presented to academic readers his research, on lectin from a snowdrop plant (Galanthus nivalis), concluding that ‘the possibility that a plant vector in common use in some GM plants can affect the mucosa of the gastrointestinal tract and exert a powerful biological effect’ (Ewen and Pusztai 1999, p. 1354). The Lancet received severe criticism from the Biotechnology and Biological Sciences Council for publishing the article, although the editor Richard Horton stood up for the publication and allegedly received ‘an aggressive call from Peter Lachmann, a former Vice-President and Biological Secretary of the Royal Society and President of the Academy of Medical Sciences (Flynn and Gillard 1999). The scientists’ reviews varied from rather sympathetic, justifying messages that Pusztai’s aim was simply to show the necessity for careful testing (Rhodes 1999) to a critique by John Pickett of Rothamsted Research, Harpenden, UK who publicly denounced the journal for ignoring his advice to reject the paper (Enserink 1999).

Biologists, such as Nina Fedoroff published their critique of his research, blaming it for serious methodological flaws (Fedoroff 2011, Fedoroff and Brown 2004). At this point, to respond to the critique, Puzstai moved to the environmental movement and published his open letter from the website of the anti-GM crops NGO Lobbywatch. His response was based on an explanation of how his case was mistreated by the Royal Society and the Rowett Institute. He provided further scientific details of his study and included a harsh personal critique of Nina Fedoroff to whom he returned the accusation of ‘superficiality in scientific matters’ (Pusztai 2006).

Importantly, before this incident Pusztai was not an activist or an official member of any civil group. He, in his own opinion, was ‘strictly science-based’, without ideology (Randerson 2008).

In April 1999 the Royal Society set up a Working Group to review its statement on GM plants for food use. It consisted of five prominent scientists. The purpose of the review was to clarify the ongoing debate on the safety of GM food started by Pusztai and to assist the Royal Society in developping its stance on the debate. Members of the Group requested six independent reviewers across different disciplines to provide their assessment of the study. They also contacted the author to request additional data, which he had indicated existed but never provided these on the grounds that these were internal documents (Royal Society 1999, p. 2).

The Group did not find ‘convincing evidence of adverse effects from GM potatoes’ as the study had ‘technical limitations of the experiments and the incorrect use of statistical tests’ (ibid. p. 1). The report reiterated the previous Royal Society statement that ‘any over-arching body analyse the current regulations, giving particular consideration to whether long-term animal feeding studies are necessary to provide greater information on allergenicity or toxicity’ and recommended that any study on GM food safety should be peer reviewed before being published (ibid., p. 5).

After his ‘150 s of TV “fame”, as he put it himself, Dr Pusztai remained in the GM debate but kept a lower profile. In 2008, in a letter to anti-GM activists, he wrote:

I asked for a credible GM testing protocol to be established that would be acceptable to the majority of scientists and to people in general. 10 years on we still have not got one. Instead, in Europe we have an unelected EFSA^3^ GMO Panel with no clear responsibility to European consumers, which invariably underwrites the safety of whatever product the GM biotech industry is pushing onto us. All of us asked for independent, transparent and inclusive research into the safety of GM plants, and particularly those used in food. There is no much sign of this either. There are still ‘many opinions but very few data (cited in GMWatch 2009).

In 2008 he insisted he was ‘not a campaigner’ or a member of any lobbying group (Randerson 2008). Yet he received, together with his wife, the Stuttgart Peace Prize, the annual award by the German NGO “Die AnStifter” (the Instigators) to people or projects involved “in a special way for peace, justice and world solidarity”. The news of award was announced by another NGO GMWatch (GMWatch 2009).

The Pusztai affair is so significant not only because it catalysed the debate over the use of GM crops but it also provoked another debate about the very way scientific experiments are conducted, interpreted and communicated. The debate moreover went beyond the borders of Britain, and into the countries with a natural suspicion of GMOs where it was well received, as the German award to Dr Pusztai shows. One can argue that the impacts of the Pusztai affair can be found over a long-term period at the European level. The European authorities adopted the Novel Foods Regulation in 1997 which also covered GM food. However, this was ‘largely inoperable: it did not contain specifics on implementation, and it was left to individual member states to define thresholds, testing methods, products subject to testing and the content of labels’. This led national governments to adopt their own measures. Austria initiated the process, by being first to ban GMOs in 1997 followed by Luxemburg, France, Greece and Germany (van der Heijden 2010).

### Case Study 2: The Seralini Case

Another example of the biotechnology ‘advocacy science’ is the Seralini case. Gilles-Éric Séralini, who is a professor of molecular biology at the University of Caen, France, chairs the scientific board of the organization CRIIGEN (Committee of Independent Research and Information on Genetic Engineering), which is technically an NGO.

Seralini and his research came into the light of public attention in 2012. The article ‘Long term toxicity of a Roundup herbicide and a Roundup-tolerant genetically modified maize’ was published in Food and Chemical Toxicology in November 2012 (Seralini et al. 2013). The article was retracted ‘after a thorough and time-consuming analysis of the published article and the data it reports’ (Elsevier 2012).

In March 2013 Seralini and his co-authors wrote their answers to critics in the same journal, where they repeated their arguments and accused their reviewers of being biased in the GM debate: for either working as plant biologists developing GM plants’ patents or being paid by Monsanto (Seralini et al. 2013). The article was republished in 2014 in Environmental Sciences Europe (Casassus 2014).

The Seralini study looked at the possible health effects of a Roundup-tolerant NK603 GM maize on a population of rats. The presented results suggested high toxic effects of the GM food given to the rats, including marked kidney deficiencies and high death rates (Seralini et al. 2012). The publication provoked an academic debate, resulting in eighteen letters to the editor of the journal and the original article being retracted. While the first letters presented a critique of the Seralini study from the methodological points of view (Ollivier 2013), the later were not about the study itself, but spoke more about the social impacts of the study including accusations of the scientific community having links to Monsanto (John 2014) and concerns of some scientists over ‘a manipulation of the scientific process to achieve activist gains’ (Folta 2014). Interestingly, both authors, Brian John and Kevin Folta, are both scientists and activists but on opposite sides of the GM debate.

This is the greatest difference between the two case studies: Pusztai’s publication was mostly criticized from the methodological point of view, Seralini was situated in the discussion of where the moral aspects of science and scientific communication stands. Thus, one can possibly argue that in a relatively short period of time 1998-–2012 a large change in the perception of science had happened: it was now seen in a postmodernist style as a social construct, including the natural scientists themselves.

The Seralini team delayed publication in order to match the release with the video entitled ‘Are we all guinea pigs?’, using similar rhetoric to Pusztai, and organised a media conference after which photographs of mice’s tumours appeared everywhere, including on American television (Arjo et al. 2013). As a result, the research received enormous cover. Interestingly, before receiving the paper by Seralini et al., journalists had to sign a non-disclosure agreement barring them from contacting any independent expert before publication (Lipponen 2012).

As in the Pusztai case, when the Royal Society set up a working group to reassess the contested study, in 2012 the European Commission asked EFSA to review the Seralini study. EFSA’s internal task force chaired by the Director of Regulated Products found the research ‘to be inadequately designed, analysed and reported’, criticizing its authors in providing ‘a limited amount of relevant additional information in their answer to critics published in the journal Food and Chemical Toxicology’ (EFSA 2012).

To promote their research, Seralini and his supporters created a website called GMO Seralini^4^ which is ‘owned and maintained by a group of concerned citizens and scientists’ (GMO Seralini 2015). The managing editor is Claire Robinson, the already mentioned colleague of Michael Antoniou, who also maintained contact with Aprad Puzstai. The editor of Sustainable Pulse’s GMO News website, linked to the website is Henry Rowlands, another British anti-GM crops activist. The website is supported by NGOs CRIIGEN, Open Earth Source and GMO Evidence (ibid.).

As in the case of the Pusztai study, the outcomes of the Seralini study were significant and soon spread throughout France. Some even argued that inspired by the Seralini presentation, activists destroyed a GM soybean consignment in France in 2012. Russia and Kazakhstan put a ban on imports of the GM maize used in the Seralini study, while Kenya banned imports of all GM food and Peru put a 10 year moratorium on GM crops (Arjo et al. 2013, p. 256).

Unlike Puzstai, who insisted on being ‘strictly science-based’ and without ideology, Seralini had developed his ideology before his research came into the public eye. He published several books in French which explain his views on GMOs to the general public. They are arguably examples of advocacy science. These are *Genetiquement Incorrect* (Genetically Incorrect) (2003), *Ces OGM qui Changeant le Monde* (These GMOs that change the world) (2004), and *Pesticides*-*OMG*-*Aliments Nous pouvons nous depolluer* (Pesticides-GMOs-Food. We can delete pollution) (2010).^5^

‘*Après nous le déluge*?’ (After us the Deluge?) is co-authored with a botanist, Jean-Marie Pelt who conducted his fieldwork in Western Africa and Southern Asia. The authors claim to be of ‘the few disperse voices on all continents to fight against the massacre on the living beings’ who want to give ‘our concrete scientific experience so that you judge the situation’. ‘The situation’ they refer to is that ‘humans are soon to be incapable to pass over the planet in good health’, soon to lose fertility ‘because of genetic changes’ and their status of citizenship to manage ‘their own simple lives of a human’ (Seralini and Pelt 2008, p. 9). They also mention climate change, loss of biodiversity and use of natural resources. In their opinion, to reverse the situation, humanity should face a radical transformation ‘to return to satisfactory sanitary state’ of the planet (ibid. p. 10). GM crops are presented as a biodiversity threat able to kill wildlife (ibid. p. 77).

They ask scientists, particularly biologists, to review their role, which would arguably lead to merging science with activism:

For forty years biologists played a very small role. Forget the man at the top of assembled cells, ignoring the landscape in which he moves, ignoring the planet in which he has a niche with plants and animals. Outside the research, the world is faded. He does not study anything but genes, micro- and nanoparticles. Some scientists continue to project their fantasies of simplicity, like one gene = one protein = one function (ibid. p. 13).

They refer to reductionism in biology. In addition to scientists–reductionists, they have identified another type of contemporary scientists – ‘the interventionists are those who by their own will change the state of nature—do not appear to regard the state of nature as a result of their ethics, it often does not interest them’ (ibid. p. 78).

Although they do not claim science to be ‘good or bad’, they do ‘judge the tree by its fruits’. So they criticize the post-industrial science for ‘establish[ing] a new religion’, which is based upon ‘political and economic powers’ and disapprove of ‘scientists who are not in agreement of the impact of their activities paralysed by political will or comforted by their inertia’ (ibid. p. 78). They question the moral authority of political leadership that approve GMOs and compare it with the nuclear power sector (ibid. p. 15).

As Seralini had expressed his negative views on GMOs prior to his study, this raises concern about bias in his participation and conduct of the GM research. His publications call upon actions of the general public and are examples of political activism. While it is possible to assume that the Pusztai case was accidental, the Seralini case was probably not. It could be due to conscious choice reflecting the social values of the scientist, and engagement with NGOs was not a last option but a thought-through strategy to promote his study.

Nielsen (2001) has suggested not mixing scientific research skills and personal values. He is right, but it seems difficult in practice. But in the postmodern context of uncertainty alleged about facts, different epistemologies and disagreement about values, when scientists get involved in a public debate or publicly share their professional findings and personal values, this is picked up by lobbying groups, it becomes a big storm. In the two cases analysed cases both scientists have lost at least some of their scientific credentials but gained influence among activists. A contrasting case is the one of Professor Anne Glover, whose position as a chief scientific adviser to the President of the European Commission was terminated after lobbying by NGOs in Brussels. This happened most probably because of her support for GM crops, as she said that according to the scientific consensus GM crops were safe (Briggs 2015). Unlike Pusztai and Seralini, Glover lost her position to influence public policy as a result of political lobbying but she retained her academic credentials.

## Conclusions

This paper argues the benefits of using the term “advocacy science” as synonym for activist science. It should be distinguished from citizen science in the analysis of the current debate over the role of science and its political discourse, particularly for the biotechnology science. The presence of different ‘sciences’ suggests a major change in the political and institutional context for science – its postmodern differentiation. A brief review of the historic changes at the institutional level points out that after a period of state-led public science, academic science evolved into two major hybrid forms: business science driven by the market and corporate interests and civil society science led by the civil society activism, also called citizen science. Business or corporate science has been heavily criticized by activists and to overcome such biases as profit motives and intellectual property rights greater public engagement in corporate science has been called (Guera 2009). But citizen science, particularly in its form of activist science led by NGOs is also not free of bias. They are both biased in their own different ways.

Citizenship science and activist science or advocacy science can be accepted as different concepts, although they address the same issue, the enhanced engagement of the public with science.

Such an active involvement of civil society in the discussion of bioscience, and science generally, has brought both positive and negative results and raised concerns for public policy. The BSE crisis and the Puzstai affair have questioned how policy-makers should manage risks arising, both real and imagined ones. On the one hand, this has brought general public mistrust about policymakers’ ability to assess these risks and, on the other, there has been a call for more transparency in decision-making and more open information for the general public. In Europe, policy-makers at all three levels, transnational, national and local, have organised exercises to include the public’s opinions in the debate on GM crops. This has included several opinion polls (Gaskell et al. 2003) and public debates on GM crops (e.g. ‘GM Nation?’ in the UK). These and the intensified communication between policy-makers, scientists and general public, including civil society organizations, is considered to be a democratic process (Pellegrini 2010).

At the same time, these public engagement activities raise a number of concerns, on how this public dialogue is shaped and who takes part in it (Sturgis 2014). A dialogue which is two-dimensional and does not include everyone but is run by small groups ‘might result in recommendations which diverge from the preferences of the wider population in potentially significant ways’ (ibid. p. 40).

It may look convincing that the GM crops debate is another co-option of the public debate by powerful interest groups (Jasanoff 2014). One may argue that this is an example of the abuse of the opportunities from associational democracy by a particular group of NGOs involved in the GM debate. Describing the BSE crisis, Jasanoff introduced ‘an unsettling phenomenon’ of ‘civic dislocation’ which is ‘a mismatch between what governmental institutions were supposed to do for the public and what they did in reality’ (Jasanoff 1997, p. 223). The state failed in its capacity to reassure the general public about the health risks and people started to look to other institutions (ibid.).

The concept of ‘civic dislocations’ may be linked with an argument about the modern deconstruction of a general science discourse into local narratives, especially in the context of the uncertainty and complexity of modern biotechnology research. This would explain why there are opposing opinions about the same scientific research among scientists themselves, who, one would expect, receive similar academic training and operate in the same critical milieu, as well as among activists. The Pusztai and Seralini cases provide an illustration.

Another change that is produced by postmodern science is in its channels and style of communication of scientific knowledge. The traditional way of communicating research findings through publications in an academic journal in a strictly scientific language has now been accompanied by more general public channels, such as the media and internet. These have become an option for retreat, as shown in the two cases, when it is not possible to win a debate in the academic domain. It is possible to identify three genres of such public oriented communication in the GM debate: public letters, public reports and public debates, both written and oral. All are different from an academic peer-reviewed publication read by a small group of experts in the field.

The question that remains open is what those scientists who do not want to be involved in either corporate science or advocacy science should do. With two partial institutional actors, business and civil society, the only actor remaining to provide an option for conducting less socially biased research is the state. It does look as if public—i.e. state—supported research has the greatest credibility and is considered less biased in the eyes of the public and scientific communities, particularly in the biotechnology field (Bhartnagar Mathur 2015). Given the limited public funding available, it is not feasible that public research would dominate in the GM debate. However, this should be taken into account by science policy-makers.

For the modern generation of scientists to be able to carry on their research they are now required to go out of the laboratories and practice their PR and marketing skills to be able to keep up the debate in the public domains. Some scientists have already used the same techniques as their advocacy science counterparts and started to lobby for their interests to conduct research with similar tactics to those of the activists. Arguably, they have started to apply advocacy science by advocating for science. In 2014 the Guardian newspaper has published an open letter by a group of the most reputable world plant scientists pleading with the European leaders to reverse their policy on GM crops, defending their professional credibility and demanding adequate funding for future research (Baldwin et al. 2014).

This leads this paper to conclude that the controversies in the GM debate have contributed much not just to policy-making for GM crops, but also to the way biotechnological science, and perhaps one may even say science in general, is communicated and perceived. Today science is perceived not as commonly agreed knowledge, as a universal episteme, but it has become a political discourse deconstructed in several local narratives used by different lobbying groups to promote their interests and even scientists themselves have been questioned in their role of knowledge creators and assessors of the reliability of scientific knowledge and have become equated to competing advocacy groups.

## Footnotes

1 See for example Risse-Kappen (1995), Della Porta and Tarrow (2004).

2 In 2015 Claire Robinson’s name disappeared from the website of Open Earth Source.

3 The European Food Safety Authority.

4
http://www.gmoseralini.org.

5 Amazon.com book search as 20.08.2015.

## References

[CR1] Antoniou M, Fagan J, Robinson C (2012). GMO Myths and truths. An evidence-based examination of the claims made for the safety and efficacy of genetically modified crops.

[CR2] Antoniou M, Fagan J, Robinson C (2014). GMO Myths and truths.

[CR3] Arjo G, Portero M, Pinol C, Vinas J, Matias-Guiu X, Capell T, Bartholomaeus A, Parrott W, Christou P (2013). Plurality of opinion, scientific discourse and pseudoscience: An in depth analysis of the Seralini et al. study claiming that Roundup TM Ready corn or the herbicide Roundup TM cause cancer in rats. Transgenic Research.

[CR4] Baldwin, I. T., Baulcombe, D. C., Buchmann, N., Chase, M. W., Fernie, A. R., Foyer, C. H., et al. (2014). *Genetically modified crops: An open letter to Europe*. The Telegraph.

[CR5] Bashford A (2015). Eugenics and neo-Malthusian: Infant life and death in the early twentieth century. Lecture for eugenics: Critical historical and ethical reflections.

[CR6] Baulcombe, D. (2014). *Interview*. Cambridge.

[CR99] Beck U (1992). Risk society: Towards a new modernity.

[CR7] Bhartnagar Mathur, P. (2015). *Interview*. Hyderabad.

[CR8] Bora A, Hausendorf H, Bora A, Hausendorf H (2010). Governing technology through public participation. Democratic transgressions of law: Governing technology through public participation.

[CR9] Briggs, H. (2015). *Ex-EU science chief adviser Anne Glover: GM tech ‘is safe’*, BBC News.

[CR10] Casassus, B. (2014). *Paper claiming GM link with tumours republished*. Nature News.

[CR11] Casetta E, Tambolo L, Michaud N (2013). That frightening Frankenmetaphor!. Frankenstein and philosophy.

[CR12] Cohn J (2008). Citizen science: Can volunteers do real research?. BioScience.

[CR13] Crick F (1966). Of molecules and men.

[CR14] Della Porta D, Tarrow S (2004). Transnational protest and global activism.

[CR15] Derry GN (1999). What science is and how it works?.

[CR100] Edwards M, Fowler A (2002). The Earthscan reader on NGO management.

[CR16] EFSA. (2012). Statement of EFSA. Final review of the Séralini et al. (2012a) publication on a 2-year rodent feeding study with glyphosate formulations and GM maize NK603 as published online on 19 September 2012 in Food and Chemical Toxicology. *EFSA Journal, 10*(11), 2986.

[CR17] Elsevier. (2012). *RETRACTED: Long term toxicity of a Roundup herbicide and a Roundup-tolerant genetically modified maize.* http://www.sciencedirect.com/science/article/pii/S0278691512005637 as viewed 20.08.2016.

[CR18] Enserink M (1999). Transgenic food debate. The lancet scolded over Pusztai paper. Science.

[CR19] Ewen SW, Pusztai A (1999). Effect of diets containing genetically modified potatoes expressing *Galanthus nivalis* lectin on rat small intestine. Lancet.

[CR20] Fagan, J. (2007). *A science-based, precautionary approach to the labelling of genetically engineered foods*. Paper by Dr. John Fagan. http://www.psrast.org/jflabel.htp. Accessed June 23 2014.

[CR21] Fairclough N (1995). Critical discourse analysis.

[CR22] Fairfield P (1994). Habermas, lyotard and political discourse. Reason Papers.

[CR23] Fedoroff, N. (2011) *Pusztai’s potatoes—Is ‘Genetic Modification’ the Culprit?* AgBioWorld. http://www.agbioworld.org/biotech-info/articles/biotech-art/pusztai-potatoes.html as viewed 20.08.2015.

[CR24] Fedoroff N, Brown NM (2004). Mendel in the kitchen: A scientist’s view of genetically modified foods.

[CR25] Feld WJ (1972). Nongovernmental forces and world politics: A study of business, labor, and political groups.

[CR26] Flynn, L., & Gillard, M. S. (1999). *Pro-GM food scientist ‘threatened editor’*, the Guardian.

[CR27] FOET. (2013). *Jeremy Rifkin*. http://www.foet.org/JeremyRifkin.htm as viewed 20.082015.

[CR28] FOET. (2013). *Pure food campaign*. http://www.foet.org/past/PureFoodCampaign.html as viewed 20.08.2015.

[CR29] Folta K (2014). Letter to the editor. Food and Chemical Toxicology.

[CR30] Forbes, P. (2011). *The God Species by Mark Lynas—Review.* The Guardian.

[CR31] Free GM (1999). Pusztai potatoes: The chernobyl of biotech, GM-Free.

[CR32] Gaskell, G., Allum, N., & Stares, S. (2003). *Europeans and biotechnology in 2002: Eurobarometer 58.0*.

[CR33] Glover, D. (2002) Transnational corporate science and regulation of agricultural biotechnology. In *Economic and political weekly*.

[CR34] GMOSeralini. (2015). *About*. http://www.gmoseralini.org/about-us/ as viewed 20.08.2015.

[CR35] GMWatch. (2009). *Pusztai to receive stuttgart peace prize*. http://www.gmwatch.org/news/archive/2009/11801-pusztai-to-receive-stuttgart-peace-prize as viewed 20.08.2015.

[CR36] Griekspoor, P. J. (2014). *Checking up on Open Earth Source’s anti-GMO stance*, Farm Progress. http://farmprogress.com/blogs-checking-earth-open-sources-anti-gmo-stance-8549 as viewed 20.08.2015.

[CR37] Grundmann, R. (2011). Transnational policy networks and the role of advocacy scientists: From ozone layer protection to climate Change, in global activism reader. In L. Luc Reydams (Ed.), *Continuum*.

[CR38] Guera JM (2009). Response. Bioethics at stake: The challenge of corporate science and biocapitalism. International Journal of Feminist Approaches to Bioethics.

[CR39] Habermas J (1984). The theory of communicative action vol. 1, reason and the rationalization of society.

[CR40] Hamilton, J. (1993). *Who’s afraid of Jurassic Park? Biotech ought to be*. Bloomberg Business.

[CR41] Harremoes, P., Gee, D., MacGarvin, M., Stirling, A., Keys, J., Wynne, B., et al. (2002) *The precautionary principle in the 20th century. Late lessons from early warnings*. London: Earthscan.

[CR42] Harries-Jones P (1991). Making knowledge count: Advocacy and social science.

[CR43] Hounshell DA (1988). Science and corporate strategy du Pont R&D 1902–1980.

[CR44] Howard E, Davis AK (2015). Investigating long-term changes in the spring migration of monarch butterflies (Lepidoptera: Nymphalidae) using 18 years of data from journey north, a citizen science program. Annals of the Entomological Society of America.

[CR45] Irwin A (1995). Citizen science.

[CR46] Jasanoff S (1997). Civilization and madness: The great BSE scare of 1996. Public Understanding of Science.

[CR47] Jasanoff S (2014). A mirror for science. Public Understanding of Science.

[CR48] John B (2014). Letter to the editor. Food and Chemical Toxicology.

[CR49] Kingsbury N (2009). Hybrid: The history and science of plant breeding.

[CR50] Koechlin, F. (2005). *Workshop A1—Basics of GM Technology*. http://www.blauen-institut.ch/s2_blue/pg_blu/pf/a_f.html. Accessed April 13 2016.

[CR101] Lee K., & Tyler R. (1999). International scientists raise concerns over genetically modified food. British Labour government rushes to defend biotech industry. World Socialist Web Site. 17 February 1999. http://www.wsws.org/en/articles/1999/02/food-f17.html.

[CR51] Lewidow, L. (2010) Democratizing agri-biotechnology? European Public Participation in Agbiotech Assessment. In A. Bora, & H. Hausendorf (Eds.), *Democratic transgressions of law: Governing technology through public participation* (pp. 75–99). Boston: Brill.

[CR52] Lewontin RC, da Costa K. Philip B (2008). Interview with Richard Lewontin. Tactical biopolitics. Art, activism and technoscience.

[CR53] Lipponen, S. (2012). *EUSJA Statement on embargoes and manipulation*. European Union of Science Journalists Association. http://www.eusja.org/eusja-statement-on-embargoes-and-manipulation/ as viewed 20.08.2015.

[CR54] Looijen, R. C. (2000). *Holism and reductionism in biology and ecology. The mutual dependence of higher and lower level research programmes*. Episteme vol. 23. Dordrecht: Springer.

[CR55] Lubow, A. (1977). *Playing God with DNA*, New Times. 11661390

[CR56] Martineau B (2001). First fruit. The creation of Flavr Savr and the birth of biotech food.

[CR57] Mellanby K (1992). The DDT story.

[CR58] Muller Hill B, Bayertz K, Porter R (1998). The different faces of science: Is genetics a social construct?. From physico-theology to biotechnology: Essays in the social and cultural history of biosciences: A festschrift for Mikulas Teich.

[CR59] Narochnizkaia, N. A. (2008). Demokratia XXI veka: Pererozdenie smyslov I zennostey, n Oranzevye Seti. Ot Belgrada do Bishkeka, ed. by Narochnizkaya N. Moscow: Alteya.

[CR60] Nielsen LA (2001). Science and advocacy are different—And we need to keep them that way. Human Dimensions of Wildlife.

[CR61] Nowotny H, Scott P, Gibbons M (2002). Rethinking science: Knowledge and the public in an age of uncertainty.

[CR62] Ollivier L (2013). Comment on “Séralini, G.-E., et al., Long term toxicity of a Roundup herbicide and a roundup-tolerant genetically modified maize. Food and Chemical Toxicology.

[CR63] OpenPlant. (2016). *Data sharing.* http://openplant.org/research/data-sharing/ as viewed 07.07.2016.

[CR64] Peacocke A (1996). From DNA to Dean.

[CR65] PEALS (2003). The people’s report on GM.

[CR66] Pellegrini G, Bora A, Hausendorf H (2010). Biotechnologies and communication: Participation for democratic processes. Democratic transgressions of law: Governing technology through public participation.

[CR67] Pierson, S. (2012). *Science advocacy: What is it and what is the role of professional societies?* Amstanews.

[CR68] Pusztai A (1991). Plant lectins.

[CR69] Pusztai, A. (2006). *Pusztai replies to Fedoroff*. Lobbywatch.org. 14.03.2006. http://www.lobbywatch.org/archive2.asp?arcid=6338 as viewed 20.08.2015.

[CR70] Randerson, J. (2008). *Arpad Pusztai: Biological Divide*. The Guardian.

[CR71] Rao H (2009). Market rebels. How activists make or break radical innovations.

[CR72] Rhodes JM (1999). Genetically modified foods and the Pusztai affair. BMJ.

[CR73] Riesch H, Potter C (2014). Citizen science as seen by scientists: Methodological, epistemological and ethical dimensions. Public Understanding of Science.

[CR74] Rifkin J (1999). The biotech century. Harnessing the gene and remaking the world.

[CR75] Risse-Kappen, T. (Ed.) (1995). *Bringing transnational relations back in: Non-state actors, domestic structures and international institutions*. Cambridge: Cambridge University Press.

[CR76] Royal Society. (1999). *Review of data on possible toxicity of GM potatoes*. London. http://royalsociety.org/~/media/Royal_Society_Content/policy/publications/1999/10092.pdf as viewed 20.08.2015.

[CR77] Runkle D, Frankel MS (2012). Advocacy in science. Summary of a workshop convened by the American Association for the Advancement of Science.

[CR78] Russell B (1952). The impact of science on society.

[CR79] Salant W (1920). Science and the state. The Scientific Monthly.

[CR80] Sarewitz, D. (2012). *Science advocacy is an institutional issue, not an individual one*. Draft. AAAS.

[CR81] Schurman R, Munro WA (2010). Fighting for the future of food. Activists versus agribusiness in the struggle over biotechnology.

[CR82] Schwarz, E. (2010) *Interview with Evgeny Schwarz*. Moscow.

[CR83] Seralini GE, Clair E, Mesnage R, Gress S, Defarge N, Malatesta M, Hennequin D, Spiroux de Vendomois J (2012). Long term toxicity of a roundup herbicide and a roundup-tolerant genetically modified maize. Food and Chemical Toxicology.

[CR84] Seralini GE, Mesnage R, Defarge N, Gress S, Hennequin D, Clair E, Malatesta M, Spiroux de Vendomois S (2013). Answers to critics: Why there is a long term toxicity due to a Roundup-tolerant genetically modified maize and to a Roundup herbicide. Food and Chemical Toxicology.

[CR85] Seralini GE, Pelt JM (2008). Après nous le déluge?.

[CR86] Skjelsbaek, K. (1971). The growth of international nongovernmental organizations in Europe. *International Organization, Transnational Relations and World Politics, 25*(3), 420–442.

[CR87] Smil, V. (1999). *Detonator of the population explosion*. Millenium Essay, Nature, Vol. 400.

[CR88] Sturgis P (2014). On the limits of public engagement for the governance of emerging technologies. Public Understanding of Science.

[CR89] Turney J (1998). Frankenstein’s footsteps: Science, genetics and popular culture.

[CR90] van der Heijden, H. A. (2010). *Social movements, public spheres and the European Politics of the Environment*. Green Power Europe? Basingstoke: Palgrave MacMillan.

[CR91] Van Dijk, T. (1995). What is political discourse analysis. In J. Blommaert, & C. Bulcaen (Eds.), *Political linguistics*. (pp. 11–52). Amsterdam: Benjamins, 1997.

[CR92] Van Dijk T, Tannen D, Schiffrin D, Hamilton H (2001). Critical discourse analysis. Handbook of discourse analysis.

[CR98] Weber M (1962). Basic concepts in sociology.

[CR93] Weinberg S (2016). To explain the world. The discovery of modern science.

[CR95] WWF. (2015). *The evolution of WWF*. Gland: WWF. http://wwf.panda.org/who_we_are/history/50_years_of_achievements/ as viewed 20.08.2015.

[CR96] Zelko F (2013). Make it a green peace! The rise of countercultural environmentalism.

[CR97] Ziman J (1996). ‘Postacademic Science’: Constructing knowledge with networks and norms. Science Studies.

